# Action Mechanism of Essential Oils or Their Components Against Pathogenic Bacteria: Literature Review

**DOI:** 10.1155/ijm/7468450

**Published:** 2026-04-21

**Authors:** Mohamed A. El-Sakhawy, Abd El Raheim Mohammed Donia, Tawfiq N. Juraybi, Abeer Ali El-Sherbiny Ateya, Wadah Osman, Amr A. Abd-Elghany, Mohamed Mostafa Soliman, Abdel Naser A. Kobisi, Mohamed A. Balah

**Affiliations:** ^1^ Department of Medical Laboratory, College of Applied Medical Sciences, Prince Sattam bin Abdulaziz University, Al-Kharj, 11942, Saudi Arabia, psau.edu.sa; ^2^ Department of Medicinal and Aromatic Plants, Desert Research Center, El-Mataria, Cairo, Egypt, drc-egypt.org; ^3^ Department of Pharmacognosy, College of Pharmacy, Prince Sattam bin Abdulaziz University, Al-Kharj, 11942, Saudi Arabia, psau.edu.sa; ^4^ Radiology and Medical Imaging Department, College of Applied Medical Sciences, Prince Sattam bin Abdulaziz University, Al-Kharj, 11942, Saudi Arabia, psau.edu.sa; ^5^ Department of Biology, College of Science, Jazan University, Jazan, 45142, Saudi Arabia, jazanu.edu.sa; ^6^ Department of Plant Protection, Desert Research Center, El-Mataria, Cairo, Egypt, drc-egypt.org

**Keywords:** antibiotic resistance, bacteria, constituents, essential oil, mechanism of action, plant-derived active metabolites, synergism, volatile oil

## Abstract

Essential oils (EOs), derived from medicinal and aromatic plants, are natural mixtures of volatile, oily compounds with varying compositions and antibacterial mechanisms. Understanding the mechanisms of EOs and their components (EOCs) is essential for predicting their impacts on various microbes and their interactions with antibiotics. This understanding is the key to developing effective strategies to combat and overcome bacterial resistance. This review employs a thematic synthesis of research, first presenting the main chemical structures of EOCs before categorizing their proposed antibacterial mechanisms. The mechanisms of EOs and EOCs include membrane disruption, leading to increased permeability, inhibition of energy production, protein dysfunction, interference with nucleic acids (DNA/RNA), efflux pump inhibition and interference with other cellular processes such as virulence mechanisms (e.g., quorum sensing, biofilm formation and toxin production). A significant advantage is their ability to act synergistically with antibiotics, making them effective not only against antibiotic‐sensitive bacteria but also against resistant strains. Through these various mechanisms, EOs present a promising and effective approach to overcoming bacterial infections in an era of increasing antibiotic resistance.

## 1. Introduction

Pathogenic bacteria pose a major and ongoing threat to public health globally, causing a wide range of illnesses from minor infections to life‐threatening conditions. The severity of bacterial pathogenicity is influenced by numerous factors, including bacterial virulence mechanisms, host susceptibility and the escalating problem of antibiotic resistance [1].

The rising prevalence of multidrug‐resistant (MDR) bacteria, such as carbapenem‐resistant Enterobacteriaceae and methicillin‐resistant *Staphylococcus aureus* (MRSA), has intensified the urgent need for innovative treatment strategies [2]. The diminishing efficacy of traditional antibiotics against notorious ‘ESKAPE’ pathogens (*Enterococcus faecium*, *S. aureus*, *Klebsiella pneumoniae*, *Acinetobacter baumannii*, *Pseudomonas aeruginosa* and *Enterobacter* species) underscores this critical threat. This concerning situation has prompted the investigation of alternative antibacterial agents, with essential oils (EOs) derived from medicinal plants showing significant promise [3, 4].

In light of increasing antibiotic resistance, natural products, particularly EOs derived from plants, have demonstrated considerable potential for combating pathogenic bacteria. These complex blends of volatile compounds, typically extracted via distillation, exhibit broad‐spectrum antibacterial activity against both Gram‐positive and Gram‐negative pathogens [5].

EOs are intricate mixtures of volatile secondary metabolites produced by aromatic and medicinal plants. They can be extracted using various techniques, including distillation (at low or high pressure) of different plant parts, liquid carbon dioxide extraction, or microwave‐assisted extraction [6]. These oils function as a defence mechanism for the plant and typically contain 20 to 60 distinct bioactive components, with two to three major constituents present in high concentrations (20%–70%) [7, 8].

Significant attention focused on EOs of oregano, clove, cinnamon and rosemary. Composed primarily of aldehydes, terpenes and phenols, these EOs possess diverse biological activities, including antiviral, antifungal, antiprotozoal, antioxidant, anti‐inflammatory and anticancer properties [9–13].

Currently, an estimated 3000 EOs are known, with the global market value exceeding $23 billion USD annually. Demand for EO‐based products continues to grow in pharmaceutical, food preservation, cosmetic and various applications, driven by consumer preference for natural alternatives to synthetic chemicals [14]. These EOs with volatile compounds represent a valuable resource for developing innovative anti‐infective therapies rooted in nature and traditional medicine [15].

Although the antimicrobial proficiencies of EOs and their mechanisms of action (MOAs) have only been investigated recently, a molecular and cellular understanding of how EOs combat bacteria, particularly drug‐resistant strains, is now emerging [16, 17]. Recognizing these MOAs is essential for enhancing their efficacy, preventing resistance and facilitating clinical treatments [18]. The common mechanisms include inhibition of cell wall synthesis, disruption of plasma membrane integrity, interference with cellular energy production, damage to nucleic acids, inhibition of protein synthesis and alterations to key metabolic pathways [19].

The aim of this review is to address the critical need to prevent antimicrobial resistance (AMR) via EOs and to characterize their mechanism of action. While EOs and their constituents (EOCs) have long been known for their empirical antibacterial properties, this work moves to elucidate the precise molecular and cellular mechanisms through which their complex chemical constituents exert their effects. Understanding these mechanisms provides a scientific foundation for rationally developing EOs and EOCs into novel, multitargeted antimicrobial agents or potentiators of existing antibiotics. This strategy offers a promising approach to overcoming resistant pathogens, expanding our therapeutic arsenal and acting synergistically with conventional antibiotics. Elucidating the mechanisms of these synergistic actions can potentially restore antibiotic susceptibility to resistant strains and enhance therapeutic efficacy to overcome resistance.

## 2. Search Methodology

This review was conducted following a systematic approach to identify, select and synthesize the available literature on the action mechanisms of EOs or EOCs against pathogenic bacteria. The Preferred Reporting Items for Systematic Reviews guidelines were used as a framework to ensure transparency and reproducibility.

A comprehensive electronic literature search was performed to identify relevant studies. The search utilized scientific databases including PubMed, Web of Science Core Collection, Scopus and Google Scholar. The search strategy combined keywords and controlled vocabulary terms related to four core concepts: EOs, EOCs, MOA and pathogenic bacteria, along with their synonyms. Boolean operators (AND, OR) were used to connect these terms both alone and in combination.

The search was limited to articles published in English between September 1998 and November 2025, with a focus on the most recent years. This date range was selected to capture the most recent advancements in mechanistic studies while ensuring a manageable scope for a narrative review.

The exclusion criteria were studies with no mechanistic data, languages other than English, studies on nonbacterial pathogens, and studies focused solely on antifungal or antiviral activity.

## 3. Chemical Composition of EOs

**FIGURE 1:**
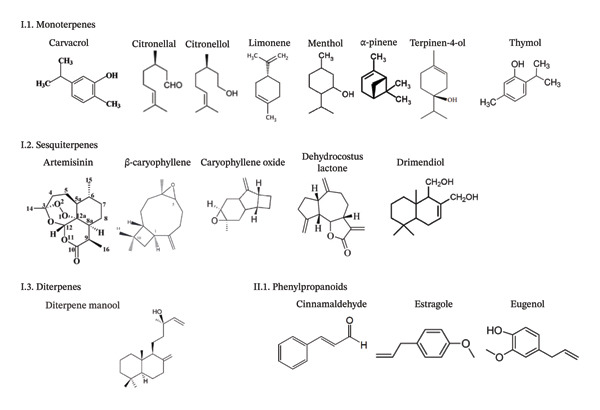
Structure of the common EOCs. This is an original figure created by the authors of this manuscript.

Derived from various primary metabolic precursors and biosynthetic routes, the constituents of EOs fall into two main categories: terpenoids (which form the bulk) and nonterpenoids, such as phenylpropanoids. These constituents are structurally diverse, consisting of hydrocarbons and their oxygenated derivatives that belong to numerous chemical families, including aldehydes, ketones, alcohols, phenols, esters, amines and sulphur compounds [20]. Figure 1 shows the structure of the common EOCs.

### 3.1. Terpenes

Terpenes are simple aromatic hydrocarbons, classified by their isoprene units, which include α‐pinene and limonene. Terpenes, a large and varied family of secondary metabolites in plants, are defined by their distinctive molecular architecture, which is based on repeating isoprene (C_5_H_8_) units. The classification of the underlying terpene structures is based on the number of carbon atoms derived from isoprene units. This results in a series: the most common are monoterpenes (C_10_), followed by sesquiterpenes (C_15_), diterpenes (C_20_), triterpenes (C_30_) and larger molecules such as tetraterpenes (C_40_). Terpenoids are oxygen‐containing hydrocarbons that have different functional groups and whose methyl groups have been oxidized, moved or removed. EOs are complex blends rich in terpenoids. These compounds include hemiterpenoids, monoterpenoids and sesquiterpenoids. EOs contain both the hydrocarbon forms and their oxygenated derivatives (e.g., alcohols and aldehydes) [20–23].

### 3.2. Monoterpenes

Monoterpenes (C_10_H_16_) are a class of terpenes consisting of two linked isoprene units, creating a ten‐carbon structure. Their structural diversity is evident in the existence of more than 30 fundamental skeletons, which are categorized into three primary subgroups (acyclic, monocyclic and bicyclic) according to their molecular configuration [20, 24]. For example, carvacrol or thymol are monoterpenoid phenols found in the EOs of several plants such as *Thymus vulgaris* [25].

### 3.3. Sesquiterpenes

As a prominent class of terpenes in EOs, sesquiterpenes (C_15_H_24_) are characterized by their three‐isoprene‐unit structure (C_15_) and lower volatility compared to monoterpenes. The presence of this extra isoprene unit grants sesquiterpenes extensive structural diversity, leading to linear monocyclic, bicyclic and tricyclic configurations [26, 27].

### 3.4. Diterpenes

Known for their chemical and structural complexity, diterpenes (C_20_H_32_) are defined by a C_20_ skeleton. Their higher molecular weight relative to monoterpenes and sesquiterpenes reduces their volatility, explaining their generally lower abundance in EOs [20, 28].

Terpenes are the most valuable compounds and the largest group of plant natural products, with a wide range of structural kinds. Although hemiterpenes (C_5_), triterpenes (C_30_) and tetraterpenes (C_40_) are also present, monoterpenes (C_10_), sesquiterpenes (C_15_) and diterpenes (C_20_) are the predominant terpenes. In addition, phenylpropane produces aromatic chemicals, which are less prevalent than terpenes. Different biochemical processes are used by plants to synthesize terpenes and the by‐products of phenylpropane; nevertheless, one primary pathway will predominate, while some may occur concurrently [6, 29, 30].

## 4. EOs’ Composition and Activity Relationship

The antimicrobial activity of EOs is fundamentally linked to their complex chemical composition. EOs are complex mixtures of volatile compounds, primarily composed of terpenes (monoterpenes, sesquiterpenes and diterpenes) and phenylpropanoids, as well as phenolics and aldehydes. Monoterpenes constitute approximately 90% of the composition of bioactive EOs [20]. EOs are synthesized through the mevalonic acid pathway in plant cells and are accumulated, produced and secreted in specialized structures such as glandular trichomes, secretory canals and other secretory structures [31].

The major antibacterial activity is displayed from (a) phenolic compounds (carvacrol, thymol, eugenol and catechol), (b) terpene hydrocarbons (p‐cymene, limonene, α‐pinene and β‐caryophyllene), (c) oxygenated monoterpenes (linalool, camphor, geraniol and terpinen‐4‐ol), (d) aldehydes (cinnamaldehyde, cuminaldehyde and citral) and (e) ketones (acetophenone and benzophenone) [32].

The antimicrobial potency of an EO depends not only on its specific composition, nature and orientation of its functional groups but also on their stereochemistry and synergistic interactions (between components) [32, 33]. The diversity of EO structure and composition enables multiple mechanisms of antibacterial action against pathogens and presents a significant advantage over conventional antibiotics that typically target specific molecular pathways [34].

## 5. EOs in Plants and Their Biological Role

Aromatic plants produce EOs, which are volatile, complex combinations of secondary metabolites that are mostly used for defence against environmental stresses, herbivores and diseases [35].

### 5.1. Ecological Functions

Ecological functions of EOs production include (a) antimicrobial defence (EOs inhibit bacterial, fungal and viral pathogens, protecting plants from infections), (b) herbivore deterrence (EOs or volatile compounds such as limonene repel insects and grazing animals), (c) pollinator attraction (floral EOs, e.g., linalool in lavender attract pollinators, enhancing reproductive success) and (d) allelopathy (EOs may suppress competing plant species by inhibiting germination or growth) [31, 36, 37]. Notably, the kind of tissue, plant species and environmental factors, including light, temperature, soil type and composition and nutrition availability, all affect the EOs’ composition and concentration [38].

### 5.2. Factors Influencing EO Yield and Composition

Factors influencing EO yield and composition include (a) environmental stress (drought or UV exposure often increases EO synthesis as a defensive response), (b) pathogen attack (fungal infections, e.g., *Fusarium* spp., can induce higher EO production in plants), (c) developmental stage (young leaves or flowers typically contain higher EO concentrations than mature tissues), (d) agricultural practices (organic cultivation and optimal harvesting times, e.g., morning for lavender, maximize EO yields) and (e) plant genetics [39–41]. Briefly, EOs provide strong antibacterial remedies for human illnesses and are essential for plant survival. Because ecological and physiological factors dynamically influence their production, standardized cultivation and extraction techniques are essential for maximizing their potential [42].

## 6. EO and EOC Mechanisms of Antibacterial Action

**FIGURE 2:**
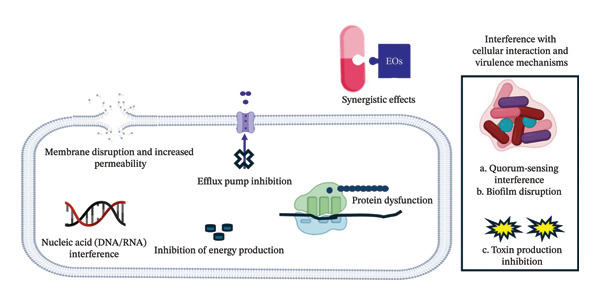
Mechanisms of EOs’ antibacterial action. This is an original figure created by the authors of this manuscript.

EOs exert antibacterial properties through multiple, often synergistic, mechanisms. These mechanisms can be categorized into three general types: suppression of virulence factors, disruption of membranes and interference with cellular functions [34]. Figure 2 presents the mechanisms of EOs’ antibacterial action. Table 1 presents the MIC values of selected common EOs and EOCs.

**TABLE 1:** MIC values of selected common essential oils (EOs) and their components.

Crude essential oil (scientific name of plant source)	Major components (%)	Sensitive bacterial pathogens (MIC values)	References
Cinnamon bark (*Cinnamomum zeylanicum*) EO	Cinnamaldehyde (57.97%)	Cinnamon bark EO MIC (6.25 μg/mL) and cinnamaldehyde MIC (2.5 μM) against *Porphyromonas gingivalis*	[43]

Clove (*Syzygium aromaticum*) EO	Eugenol (96.35%)	Carbapenem‐ and polymyxin‐resistant *Klebsiella aerogenes* (MIC = 17.57 μg/mL)	[44]

Laurel (*Nectandra megapotamica*) EO	Caryophyllene oxide (22.3%)	Multidrug‐resistant OXA‐23‐producing *Acinetobacter baumannii* (MIC 36,000 μg/mL)	[45]

Lemongrass (*Cymbopogon citratus* L.) EO	Myrcene (57.52%)	*Pseudomonas aeruginosa, Klebsiella pneumoniae* and *Staphylococcus epidermidis* (MIC range: 0.1%–3.2% v/v)	[46]

Oregano (*Origanum vulgare*) EO	2‐Menthen‐1‐ol (36.33%), linalyl acetate (9.26%), terpinene‐4‐ol (9.01%), 4‐thujanol (6.33%), menthen (5.81%), sabinene (5.18%) and carvacrol methyl ether (5.14%).	*S. aureus* (1.90 mg/mL), *E. coli* (0.49 mg/mL)	[47]

Oregano (*Origanum vulgare* L.) EO	Terpenes including α‐pinene (62%–63%) and p‐cymene (21%)	MIC values ranging from 94 to 754 μg cm^−3^ air. *Acetobacter malorum* (753.6 μg cm^−3^), *Dickeya dadantii* (94.2 μg cm^−3^), *E. coli* (753.6 μg cm^−3^), *Listeria monocytogenes* (376.8 μg cm^−3^), *Pectobacterium carotovorum* subsp. actinidiae (753.6 μg cm^−3^), *P. carotovorum* subsp. carotovorum (188.4 μg cm^−3^), *Salmonella enterica* (753.6 μg cm^−3^) and *Staphylococcus aureus* (376.8 μg cm^−3^)	[48]

Oregano (*Origanum vulgare* L., wild and cultivated) EO	Monoterpenes and sesquiterpenes, including thymol (16.75%) and carvacrol (21.98%)	Oregano EOs extracted from white flowers with MICs (range: 0.5–1 mg/mL) against tested bacteria, including *Staphylococcus aureus* (MRSA), *Enterococcus faecalis*, *E*. *faecium*, *Streptococcus agalactiae*, *E. coli* and *Salmonella typhimurium*). The antibacterial activities of carvacrol are higher than of thymol (MIC values of 0.005–0.04 mg/mL)	[49]

Oregano (*Origanum vulgare*) EO	Carvacrol (84.2%)	MIC range values (0.039%–1.250%) against multidrug‐resistant *Klebsiella pneumoniae* and *Pseudomonas aeruginosa*	[50]

Tea tree (*Melaleuca alternifolia* L.) EO	Terpinen‐4‐ol (44.55%)	The diameters of the inhibitory zones were 12.33, 14, 15.67 and 24.33 mm for *Bacillus cereus* (ATCC 11778), *Staphylococcus aureus* (ATCC 25923), *Salmonella enterica* (ATCC 13076) and *Escherichia coli* (ATCC 25922), respectively.	[51]

Tea tree (*Melaleuca alternifolia*) EO	Terpinen‐4‐ol	Tea tree EO against *Legionella pneumophila* (MIC range: 0.125–0.25), tea tree terpinen‐4‐ol against *Legionella pneumophila* (MIC % v/v range: 0.06–0.125)	[52]

Thyme (*Thymus vulgaris*) EO	Thymol (45.74%)	MIC range values (0.156%–5.000%) against multidrug‐resistant *Klebsiella pneumoniae* and *Pseudomonas aeruginosa*	[50]

Thyme (*Thymus vulgaris*) EO	Carvacrol (78.83%)	Carvacrol volatile oil exhibits antibacterial activities at low MIC range (of 64 and 128 μg/mL) against *E. coli* and all carbapenem‐resistant *Acinetobacter baumannii* isolates.	[53]

Rosemary (*Rosmarinus officinalis*) EO	1,8‐cineole (17.16%), α‐pinene (16.95%) and verbenone (15.78%)	*Proteus vulgaris*, *Staphylococcus aureus* and *Klebsiella pneumoniae* (MIC range: 0.06–0.16 mg/mL)	[54]

Abbreviation: MIC, minimum inhibitory concentration.

### 6.1. Membrane Disruption and Increased Permeability

The main mechanism of action of EOs is membrane dysfunction, which, according to the lipophilic and hydrophobic nature of most EO components, allows them to partition into bacterial membranes, causing structural and functional damage. This represents the primary and most well‐characterized mechanism by which EO enters bacteria. The loss of the cytoplasmic material and the release of potassium ions from the cell cytoplasm were interpreted as signs of severe and permanent cytoplasmic membrane damage. It was hypothesized that because of their highly lipophilic nature, the EOs and their main constituents in this investigation would compromise the integrity of the membrane, allowing intracellular components to seep into the extracellular medium. Potassium ion outflow has been demonstrated in several investigations to be the initial sign of bacterial membrane degradation [55–57].

Specific mechanisms of membrane disruption action may include key aspects, including the penetration of phospholipid bilayers, increased membrane permeability, alteration of membrane fluidity and disruption of membrane potential. The hydrophobic or lipophilic compounds in EOs can dissolve in the lipid matrix of bacterial membranes due to their lipophilicity. Carvacrol and thymol, as examples of EOs, are particularly effective at penetrating both outer and cytoplasmic membranes. EOs alter the permeability of membranes for necessary cations. Components of EOs cause membranes to develop pores or channels that allow intracellular materials, including proteins, ions, ATP and nucleic acids, to seep out. Studies on propidium iodide uptake have validated this effect for a number of EOs, such as oregano and coriander oils. EOs interfere with proton motive force and electron transport chains, subsiding the electrochemical gradient essential for bacterial viability, which leads to the disruption of membrane potential. Furthermore, the addition of EOCs changes membrane viscosity, lipid–lipid interactions and phase behaviour, affecting the function of membrane‐associated proteins, which leads to alteration of membrane fluidity [58–61].

Because the presence of a complex outer membrane of Gram‐negative bacteria offers stronger defence against hydrophobic chemicals than the cell wall, which is primarily composed of peptidoglycan, Gram‐positive bacteria are often more vulnerable or susceptible to EOs than Gram‐negative species. This describes the general strategies employed by Gram‐negative bacteria to achieve lower antibiotic permeability through their outer membrane by altering porin channels. However, by producing lipopolysaccharides (LPS), certain EO components, such as carvacrol, can break down the outer membrane of Gram‐negative bacteria, making it easier for them to absorb substances. That’s why carvacrol has an unusual EOC property that has activity against Gram‐negative bacteria than Gram‐positive [25, 31, 62–66].

### 6.2. Other Essential Oils’ Mechanisms of Antibacterial Actions

Other EOs’ mechanism of antibacterial actions is interference with intracellular processes (protein dysfunction, nucleic acid interference and efflux pump inhibition) and metabolic pathways [inhibition of energy production] [6, 67]. EOs inhibit bacterial cell permeability, although the presence of additional mechanisms or targets cannot be ruled out [56].

#### 6.2.1. Inhibition of Energy Production

Numerous chemicals found in EOs either block important enzymes involved in energy metabolism or uncouple oxidative phosphorylation. Carvacrol, for instance, has been demonstrated to lower ATP synthesis in both Gram‐positive and Gram‐negative bacteria [68].

Thymol and carvacrol exert their antimicrobial effects through multiple mechanisms, including damaging cell membranes, disrupting energy metabolism, modulating oxidative stress and inhibiting efflux pumps, thus enhancing their antimicrobial effects and preventing resistance of *S. aureus* and *A. baumannii* bacteria [69].

The intracellular activity of thymol (volatile oil as one of the EO compounds) suggests that it interferes with critical energy‐generating functions, reducing a cell’s capacity to recover from thymol exposure [68]. Since energy production is required for cell recovery, inhibition of the ATPase may be crucial for cell death at high eugenol concentrations [70].

#### 6.2.2. Protein Dysfunction

EOCs can denature proteins or inhibit their synthesis. For example, cinnamaldehyde binds to FtsZ, a protein crucial for bacterial cell division in *B. cereus*, thereby inhibiting growth. Thymol and carvacrol compounds of EOs have been shown to coagulate cytoplasmic proteins [71, 72].

After being exposed to cinnamon bark EO, *E. coli* cells had a lower negative charge, and bacterial cells suffered irreversible membrane damage from the acidification and protein denaturation of the cell membrane brought on by the accumulation of the oil’s constituents, which made it possible for antibiotics to reach PBPs (penicillin‐binding proteins) and induce cell death [67, 73].

Eugenol and cinnamaldehyde, for example, can interact with proteins and enzymes to reach the mitochondria and cytoplasm, preventing the synthesis of ATP, which is essential for bacterial survival [13, 74–76]. Additionally, it has been discovered that certain elements of natural oils interfere with the efflux pump mechanism, which is produced by strains of bacteria that are resistant to many antibiotic drugs [13].

Trans‐cinnamaldehyde is one of the main ingredients of cinnamon EO. This phenylpropene may bind to proteins and interfere with membrane permeability by penetrating the phospholipid bilayer of the bacteria. Additionally, it inhibits the movement of ions and proteins, causing cytoplasmic coagulation, protein denaturation and a reduction in metabolites and ions, all of which lead to bacterial mortality. By saturating a sizable percentage of fatty acids in the phospholipid membrane, trans‐cinnamaldehyde also gives the bacterial membrane stiffness [43].

Other components in cinnamon EO, besides trans‐cinnamaldehyde, impede the electron transport chain in mitochondria, preventing the production of ATP [77]. Furthermore, it prevents the protein FtsZ (bacterial cell division protein), which controls bacterial cell division, from polymerizing in a GTP‐dependent manner [71]. According to Vasconcelos et al. [67], cinnamon EO affects the expression of bcsA and luxR, two important components of quorum sensing, the cell communication system necessary for structuring the biofilm [67].

It should be mentioned that, unlike other antimicrobials, EOs are distinguished by their capacity to enter the periplasm of Gram‐negative bacteria via the porin protein [78].

#### 6.2.3. Nucleic Acid (DNA/RNA) Interference

Some EOCs interact with nucleic acids, inhibiting replication, transcription and metabolic pathways. Eugenol from clove oil has demonstrated DNA‐binding capability [79].

The EO extracted from clove buds contained eugenol primarily. Against *S. aureus*, the EO demonstrated antibacterial activity; the antibacterial effects were contingent upon the oil’s concentration and duration of action. Additionally, the EO from clove buds enters the cell through the cytoplasmic membrane or breaks through it once the cell structure is destroyed. This prevents the normal synthesis of proteins and DNA, which are necessary for the growth of bacteria [80].

The clove EO treatment caused considerable intracellular component leakage and morphological damage to the *S. aureus* bacterial cells. By blocking the tricarboxylic acid cycle pathway, clove EO dramatically reduced *S. aureus*’s respiratory metabolism at the metabolic level. The expression levels of *S. aureus* virulence genes were significantly reduced as a result of molecular biology experiments that demonstrated clove EO could interact with bacterial DNA molecules and influence the accessory gene regulator system [81].

The growth of Enterohemorrhagic *E. coli* (O157:H7 strain) may be efficiently inhibited by the EO of *Litsea cubeba*. The EO of *L. cubeba* was found to have a strong ability to penetrate membranes in the investigation of antibacterial mechanisms. This ability might disrupt the bacterial cell structure and alter membrane permeability, which would allow intracellular organic materials to seep out. Additionally, the physiological metabolism of Enterohemorrhagic *E. coli* (O157:H7 strain), including respiratory metabolism, enzyme activity, nucleic acid replication and the transcription level of key virulence genes (stx1, stx2, ehxA and eae), is inhibited by *L. cubeba* EO [82].

Ginger EO demonstrated potent antibacterial effects on two foodborne pathogens, showing greater efficacy against *S. aureus* than against *E. coli*. Its primary mode of action involves damaging the bacterial cell membrane. This damage causes essential internal components, such as proteins and nucleic acids, to leak out. As a result, the cell’s metabolic activity declines, leading to its death [83]. At a molecular level, the treatment disrupts bacterial physiology by altering the expression of genes responsible for key enzymes involved in cell lysis. This was evidenced by a significant increase in nucleic acids outside the cell and a sharp decrease in bacterial metabolic activity. Furthermore, ginger EO was found to suppress genes related to critical bacterial functions, including energy production, the tricarboxylic acid cycle, membrane protein synthesis and DNA metabolism [83].

According to Akermi et al. [84], the EO of *T. vulgaris* strongly inhibits pathogenic bacteria by disrupting DNA replication and transcription. Molecular docking simulations identified thymol and β‐sesquiphellandrene as key compounds responsible for this antibacterial effect; they work by blocking the functions of topoisomerase II, DNA polymerase and RNA polymerase, which severely hinders bacterial genetic processes. Furthermore, VEGA‐QSAR modelling suggests that this EO is a safe source for future antibacterial agents. ADME (absorption, distribution, metabolism and excretion) analysis confirmed that these compounds adhere to Lipinski’s Rule of Five, indicating their potential as candidates to combat antibiotic resistance. Additional in silico PASS prediction studies also revealed other beneficial bioactivities and enzymatic targets within the oil, pointing to its future applicability in mitigating serious diseases [84].

#### 6.2.4. Efflux Pump Inhibition

Active efflux, a process where bacteria use membrane transporters to expel a wide range of antibiotics, is a primary mechanism of drug resistance. While these efflux pumps contribute to a bacterium’s natural low‐level resistance, the emergence of multidrug resistance in clinical settings is driven by their overproduction, the acquisition of mutations and their synergistic action with other resistance strategies. Therefore, efflux pumps are a promising target for new therapies. Inhibiting these pumps offers a strategic approach to counteract resistance by increasing intracellular antibiotic levels, thereby restoring drug efficacy. Furthermore, these inhibitors can help reduce the development of additional resistance mechanisms and serve as diagnostic tools to determine the role of efflux in a specific bacterial isolate (Bhardwaj and Mohanty, 2012) [85]. Small ion efflux is not always a sign of total membrane dysfunction; it can occur in healthy cells when growth is suppressed because the cell uses energy for survival or repair rather than for cell division [86].

The emergence of antibiotic‐resistant bacteria makes the inhibition of efflux pumps a highly promising mechanism. Research shows that EOs derived from *Salvia fruticosa*, *S. officinalis* and *S. sclarea* enhance the effectiveness of tetracycline against resistant *S. epidermidis* strains. These EOs lower the minimum concentration of tetracycline needed to inhibit bacterial growth, reduce the actual export of the antibiotic and suppress the expression of the tet(K) resistance gene. Among the tested oils, *S. fruticosa* demonstrated the strongest effects [87]. These molecular and functional analyses of the inhibitory impact, particularly of *S. fruticosa* oil on the Tet(K) pump, provide a foundation for subsequent research into pharmacokinetic and pharmacodynamic properties. Using an efflux pump inhibitor alongside an antibiotic could restore the drug’s effectiveness against bacteria that have developed efflux‐based resistance. This approach has the dual benefit of potentially improving an antibiotic’s clinical performance while reducing the likelihood of selecting for further resistant mutants [87, 88].

Certain EOCs, like trans‐caryophyllene, can block bacterial efflux pumps, counteracting a major antibiotic resistance mechanism [89]. Farnesol and geraniol, rose oil, palmarosa oil and citronella oil components inhibit *Mycobacterium smegmatis* efflux pumps [90]. Geraniol, an EO constituent, has been observed to function as an efflux pump inhibitor, hence inhibiting MDR *Enterobacter aerogenes* [87, 91, 92].

Thymol and carvacrol, as EOCs, exert their antimicrobial effects through multiple mechanisms, including inhibiting efflux pumps, thus enhancing their antimicrobial effects and preventing resistance of *S. aureus* and *A. baumannii* bacteria [69].

Common constituents of EOs extracted from aromatic plants include farnesol, geraniol, thymol and carvacrol, indicating that EOs would also provide effective sources of efflux pump inhibitors [90].

Cinnamon bark oil showed the strongest antimicrobial activity against all clinically relevant *P. aeruginosa* MDR strains. In addition, cinnamon bark oil and cinnamaldehyde combined with colistin demonstrated synergistic rates, which may be attributed to the inhibition of the efflux pump [93, 94].

### 6.3. Interference With Other Cellular Processes, Cellular Interaction and Virulence Mechanisms

Beyond the common intracellular mechanisms (listed before) of membrane disruption, intracellular protein dysfunction, nucleic acid interference, efflux pump inhibition and the impairment of energy production, EOs and EOCs can also disrupt other cellular processes, cellular interactions and virulence mechanisms.

#### 6.3.1. Quorum‐Sensing Interference

Quorum sensing is the technique by which bacteria employ the language of low‐molecular‐weight ligands to determine the densities of their populations. Gram‐positive and Gram‐negative bacteria have different kinds of quorum‐sensing mechanisms. N‐acyl homoserine lactones are the signal molecules that Gram‐negative bacteria employ the most frequently. Gram‐positive quorum sensing often uses tiny signal peptides that have undergone post‐translational processing as signalling molecules. A two‐component histidine kinase signalling system’s sensory component interacts with these Gram‐positive signalling peptides [95, 96].

Some EOCs block bacterial cell‐to‐cell communication systems, which attenuates bacterial virulence factor production [97]. It has been observed that several EOs and EO compounds, including carvacrol, limonene, eugenol, thymol and 1,8‐cineol, and cassia oil, cinnamon oil, eucalyptus oil, clove oil, tea tree oil, oregano oil and thyme oil, can alter the expression of genes involved in the production of virulence factors, autoinducer molecules and biofilms. The Gram‐negative bacterium *Chromobacterium violaceum* was used to demonstrate the antiquorum‐sensing ability of certain EOs and single EO molecules. Quorum‐sensing autoinducer molecules known as acyl homoserine lactones control the manufacture of the violet‐coloured substance known as violacein, which is produced by reporter strains of these bacteria. The finding that enantiomeric monoterpenes had distinct effects on the quorum‐sensing regulatory system was of great interest. Carvone, limonene and borneol’s (+) ‐ enantiomers promoted the development of violacein, whereas their (−) analogues prevented the synthesis of violacein [98].

*Salmonella enterica* subsp. Typhimurium, *S. aureus* tested strain and *C. violaceum* biofilm production were all inhibited by carvacrol, one of the main antibacterial components of oregano oil. Additionally, it decreased the production of violacein, chitinase activity and civil (a gene encoding for the N‐acyl‐L‐homoserine lactone synthase), all of which are regulated by quorum sensing [99].

The quorum sensing of *C. violaceum* was inhibited by oregano EO added to edible films or coatings, which also prevented intercellular communication. The pectin–oregano EO combinations also show antibacterial activity against food deterioration and harmful microbes. Studying how the treatments affect the pathogenesis of the microorganisms under investigation can benefit from these findings [100].

Antibacterial, antiquorum‐sensing and antibiofilm EO molecules offer a viable way to improve illness management, including against *Vibrio* spp. [101].

#### 6.3.2. Biofilm Disruption

A bacterial colony encased in an autogenerated polymer matrix comprising proteins, DNA and polysaccharides is called a biofilm. Because bacterial biofilms are more resilient to antibiotics, disinfectants and the body’s immune system, they result in chronic infections. A gradient of oxygen and nutrients from the top layer to the bottom layer of biofilms is another important feature of biofilms [102]. The capacity of biofilms to give resistance and tolerance to antibiotics is one of their defining characteristics. In certain pathogenic bacteria, the extracellular matrix of polymeric compounds binds antimicrobial drugs, prevents drug diffusion and promotes enzymatic inactivation [103].

The ability of *Streptococcus mutans* to form biofilms is a crucial virulence factor that underpins the pathophysiology of dental caries (tooth decay). This process is significant primarily because the biofilm’s hierarchical architecture provides a protected milieu for bacterial persistence and multiplication. Biofilm development begins when surface adhesins allow *S. mutans* to adhere to the salivary pellicle on the tooth surface. Subsequently, glucosyl transferase enzymes convert sucrose into extracellular glucans, forming a sticky polysaccharide matrix that promotes the coadhesion of a diverse microbial population. This biofilm protects the microorganisms from antimicrobial agents and host immune defences. Thus, the development of a biofilm is essential for the cariogenicity of *S. mutans*, as it transforms a temporary colonization into a chronic, disease‐causing state [104, 105].

Biofilms, which can make bacteria 10–100 times more resistant to antibacterial antibiotics, are particularly vulnerable to EO penetration due to the oils’ ability to diffuse through the extracellular matrix [103, 106].

Many EOs inhibit biofilm formation or disrupt pre‐existing biofilms of *S. mutans* at low concentrations. Oregano EO and its thymol and carvacrol components eliminate mature biofilms by changing the extracellular polysaccharides synthesis and pH value. Oregano EO has shown potent antibiofilm activity against *S. mutans* and other pathogens [107–109].

#### 6.3.3. Toxin Production Inhibition

Numerous harmful bacteria generate potentially fatal toxins that, through receptor‐mediated interactions, disrupt cellular function or cause cytotoxicity at the colonization site or other parts of the body. Toxin expression in these pathogens is influenced by a number of factors, such as biotic and abiotic settings, competing bacteria and chemical cues. According to recent research, a number of natural substances may influence the generation of toxins by harmful bacteria [110, 111].

*S. aureus* strains secrete two main virulence factors: enterotoxins and α‐haemolysin. The 33‐kDa pore‐forming protein known as α‐haemolysin possesses cytolytic, haemolytic and dermonecrotic properties. In humans, α‐haemolysin affects a variety of cells [112]. One form of food poisoning in humans is staphylococcal enterotoxins (SEs), which are the virulence factors that cause staphylococcal gastroenteritis. Additionally, the enterotoxins exhibit the immunomodulatory qualities of superantigens, promoting T‐cell activation and the production of cytokines produced from T cells [113].

Certain EOs reduce the synthesis or secretion of bacterial toxins. Some EOs inhibit enterotoxin production in some bacteria. The EO of *Origanum vulgare* L. suppresses the production of SEs and stops *S. aureus* from growing [114]. *O. vulgare* EO significantly suppressed the growth and some metabolic traits of isolated *S. aureus* strains, such as the activities of lipase, coagulase and salt tolerance [115].

EOs from clove, cinnamon, oregano, *Zataria multiflora*, eugenol and 4‐hydroxytyrosol inhibit *S. aureus* haemolysin, enterotoxin (A, B), toxic shock and syndrome toxin by the potential mechanism of action of reduced expression of toxin production genes, sea, seb, tst and hla [110]. High levels of trans‐anethole found in fennel oil demonstrated anti‐*S. aureus* activity. Fennel oil may reduce the expression of *S. aureus* exotoxins, such as α‐toxin, SEs and toxic shock syndrome toxin 1, in a dose‐dependent manner when taken at subinhibitory concentrations [116].

In both MRSA and MSSA, subinhibitory thymol concentrations reduced the synthesis of α‐haemolysin and SEs A and B (SEA and SEB) in a dose‐dependent way. α‐haemolysin and enterotoxins are expressed at subinhibitory levels when thymol is present. The structure of thymol could serve as a foundation for the creation of medications that target these virulence factors in bacteria [112].

The EO of *Syzygium aromaticum* inhibits the formation of toxins and important bacterial enzymes, both of which are needed for the virulence of many harmful bacteria. It prevented *Listeria monocytogenes* from producing the toxin known as listeriolysin O [117, 118]. Carvacrol (natural monoterpene EO) prevented the growth of vegetative bacteria and the formation of diarrhoeal toxins by *Bacillus cereus* [25].

Since bacteria are less likely to become resistant to substances that target numerous biological systems at once, EOs’ multitarget antibacterial effect makes them especially helpful in an era of rising antibiotic resistance [119].

### 6.4. Synergistic Effects

EOs are complex natural mixtures with broad antimicrobial properties, primarily attributed to oxygenated terpenoids such as phenolic terpenes, phenylpropanoids and alcohols. While some constituents (e.g., hydrocarbons) are less active on their own, they can enhance overall bioactivity in combination. The interactions between these components can be antagonistic, additive or synergistic and are commonly evaluated using checkerboard, graphical and time‐kill assays. Further research into their MOA and toxicology is needed to optimize their application [33].

The effect of two or more agents working together that is larger than the anticipated additive effect of those agents is known as synergy [124]. A synergistic pair of components is more effective together than separately, meaning a lower total concentration is required to achieve the same antimicrobial effect as the sum of the purified components acting alone [125]. Synergy in this study does not mean combination between EOs with antibiotics, EOs with other EOs, but also EO constituents with other constituents [126].

Synergistic antimicrobial interactions are commonly achieved through several mechanisms: sequential inhibition of a shared biochemical pathway, the inhibition of bacterial protective enzymes and the use of cell wall‐active agents to increase the penetration of other antimicrobials, often by disrupting the wall and inhibiting protein synthesis [33, 127].

One of the most promising aspects of EO or EOC is their ability to enhance the effectiveness of EOC or conventional antibiotics through synergistic interactions.

#### 6.4.1. EOC–EOC Synergy

The use of natural oils improves the synergistic action of phytochemicals, allowing them to work through many phases to increase antimicrobial activity, rather than isolating pure components. For example, substances containing functional groups or lipophilic substances such as terpenes and phenols can target cell membranes [13, 68, 128].

Carvacrol or cymophenol are monoterpenoid phenols found in the EO of wild bergamot, pepperwort, thyme and *O*. vulgare. P‐cymene, a natural EO monoterpene with a benzene ring and no functional groups on its side chains, is a precursor to carvacrol and is not an effective antibacterial agent on its own; nevertheless, it enhances the activity of other compounds, such as carvacrol [25].

The antimicrobial effect of carvacrol is enhanced (synergized) by other compounds. Weakly antimicrobial hydrocarbons help by breaking down the cell membrane, making it easier for carvacrol to get inside. Furthermore, when combined with eugenol, carvacrol (or thymol) is thought to break the membrane so eugenol can enter and affect proteins. A different type of synergy occurs between eugenol and cinnamaldehyde, where they probably attack different proteins simultaneously [129–131].

According to Zhou et al., the synergistic action of cinnamaldehyde combined with either thymol or carvacrol against *S. typhimurium* can be explained by two hypotheses. One possibility is that thymol or carvacrol compromises the cell membrane’s integrity, making it easier for cinnamaldehyde to penetrate the cell. Alternatively, these compounds may amplify the effect of cinnamaldehyde by stabilizing or increasing the pores it forms in the membrane, leading to greater cellular damage when used together [128].

To explain the synergy between thymol and carvacrol against *S. typhimurium*, the authors proposed four hypotheses. The compounds might (i) employ different antibacterial mechanisms, (ii) act on different bacterial targets, (iii) have similar mechanisms that are enhanced in combination or (iv) only inhibit the pathogen effectively when applied together. This disruptive effect on cell membranes was also demonstrated by other synergistic EO blends, including oregano/basil against *E. coli* and oregano/bergamot against *B. subtilis*, which all caused significantly greater membrane damage compared to untreated cells [33, 132].

#### 6.4.2. EO–Antibiotic Synergy

Antibiotic resistance generally arises through three primary mechanisms: stopping the antibiotic from reaching its target, pumping the drug out of the bacterial cell or breaking down or altering the antibiotic molecule itself [133]. The combination of plant natural products and antibiotics commonly results in enhanced antimicrobial efficacy, a phenomenon known as synergistic activity, although additive and antagonistic effects are also observed [134]. Table 2 represents the synergistic effect of selected common EOs and EOCs with antibiotics on bacteria.

**TABLE 2:** Synergistic effect of selected common essential oils (EOs) and their components with antibiotics against bacteria.

Crude essential oil (scientific name of plant source)	Major components (%)	Synergistic effect of EO/EOCs and antibiotic/EO combination (activity and MIC) against bacteria	Reference
Cinnamon (*Cinnamomum cassia*) EO	—	Cinnamon EO + ampicillin (FICI 0.38) synergy against *Staphylococcus aureus.* Cinnamon EO + chloramphenicol (FICI 0.50) synergy against *Staphylococcus aureus and E. coli.*	[120]

Ajowan (*Trachyspermum ammi*) EO	Thymol (50·75%), γ‐terpinene (25·94%) and p‐cymene (18·31%)	The most sensitive organisms to ajowan EO were *Streptococcus pneumoniae* (MIC = 0·125–0·5 mg mL^−1^). Synergistic effects were observed with crude EO + amoxicillin against *Staphylococcus aureus* (MRSA) isolate (FICI range: 0·37–0·50) and with crude EO + ciprofloxacin against *Pseudomonas aeruginosa* (standard strain)*, Staphylococcus aureus* (standard strain) and penicillin (P)‐resistant *S. pneumoniae* (FICI = 0·37–0·50).	[121]

Oregano (*Origanum vulgare*) and thyme (*Thymus vulgaris*) EOs	Carvacrol (84.2%) is the main component in oregano EO, while thymol (45.74%) is the main component in thyme EO.	Oregano EO + ceftriaxone shifting in sensitivity pattern from resistant to sensitive of KP17 (*K. pneumoniae* strain). Also, antimicrobial activity is increasing in some combinations of oregano EO + ciprofloxacin for certain *Pseudomonas aeruginosa* isolates. Tested EOs can boost conventional antibiotic activity at even low concentrations (0.1% v/v), however, more consistently in moderately sensitive bacteria.	[50]

Oregano (*Origanum vulgare* L.) EO (wild and cultivated)	Monoterpenes and sesquiterpenes, including thymol (16.75%) and carvacrol (21.98%)	Carvacrol + tobramycin gave highly promising synergistic effects against *E. coli* (FICI = 0.25) and MRSA (FICI = 0.125); the results also proven by the time‐kill activity.	[49]

Eucalyptus (*Eucalyptus globulus* Labill) EO	1,8‐cineole (58.07%), linalool (12.05%), linalyl acetate (10.95%), camphor (4.39%) and α‐pinene (2.33%).	EO + antibiotic (cefotaxime, vancomycin, oxacillin) showed a synergistic effect, while no antagonistic effects were found. Moreover, EO + antibiotic combinations, a positive modulation in the decrease in antibiotic resistance among the pathogenic strains [vancomycin‐resistant *enterococci*, *Staphylococcus aureus* (MRSA) and broad‐spectrum β‐lactamase‐producing *Escherichia coli],* the combination lowers antibiotic concentration, ranging approximately from fourfold to 512‐fold MIC against the tested bacteria *E. coli*, *E. faecalis* and *S. aureus*	[122]
Tea tree (*Melaleuca alternifolia* Chell) EO	Terpinen‐4‐ol (43.29%), γ‐terpinene (20.16%) and α‐terpinene (8.89%).		

Thyme (*Thymus vulgaris*) EO	Carvacrol (78.83%)	Synergy of thyme EO + imipenem shifts MIC of imipenem by eightfold to 16‐fold in the carbapenem (FICI< 0.5) against *Acinetobacter baumannii* isolates. Synergy of carvacrol volatile oil + imipenem against *E. coli* and all carbapenem‐resistant *A*. *baumannii* isolates (FICI < 0.5).	[53]

Thyme (*Thymus vulgaris*) EO	Limonene (59.28%) and β‐pinene (10.25%)	In vitro antibacterial activity, the binary combination (1:1 ratio) of thyme/cloves EOs is synergistic against both *E. coli* mC1(FICI = 0.630) and *Staphylococcus aureus* mC2 food isolates (FICI = 0.830)	[123]
Cloves (*Syzygium aromaticum*) EO	Caryophyllene (approximately 64.29%), eugenol (17.00%) and α‐humulene (11.57%)		

Abbreviations: FICI, fractional inhibitory concentration indices; MIC, minimum inhibitory concentration.

The combination of oregano EO and certain antibiotics (fluoroquinolones, doxycycline, lincomycin and mequindox florfenicol) demonstrates a synergistic effect against *E. coli*. For infections caused by extended‐spectrum beta‐lactamase‐producing strains, this combination may substantially lower the effective therapeutic dose of the antibiotics, which would minimize their associated side effects [135].

A study evaluating the antibacterial activities of oregano EOs, carvacrol and thymol found that the EO possessed superior efficacy against MRSA strains. Furthermore, carvacrol was identified as a more effective antibacterial compound than thymol. Synergy testing indicated that carvacrol produced stronger synergistic effects with various antibiotics, most notably tobramycin. These synergistic results were validated through time‐kill assays and an in vivo murine model, demonstrating that the carvacrol–tobramycin combination exceeded the efficacy of tobramycin monotherapy. The underlying mechanism is preliminarily suggested to involve carvacrol perforating the bacterial surface, thereby promoting tobramycin permeability across the lipid bilayer of the membrane. These findings establish a theoretical basis for the potential therapeutic use of these compounds [49].

The EO from *Aloysia gratissima* and its major constituent, β‐caryophyllene, not only exhibited their own antibacterial properties against *S. aureus* but also significantly increased the effectiveness of conventional antibiotics. They boosted norfloxacin’s action against multiple bacterial species and successfully counteracted resistance to gentamicin in *S. aureus* and to erythromycin in *P. aeruginosa*. This was confirmed by observing a lower MIC for the antibiotics when combined with the oil or β‐caryophyllene [136].

According to Dhara and Tripathi [137], cinnamaldehyde combined with cefotaxime or ciprofloxacin showed antibacterial and synergistic activity against extended‐spectrum beta‐lactamase, quinolone‐resistant (ESBL‐QR) *E. coli* and *K. pneumoniae*. This confirms cinnamaldehyde’s therapeutic value against these resistant bacteria. The synergism with cefotaxime suggests that the compound has a potent mode of action, both independently and as an adjunct to antibiotics [137].

Research indicates that eugenol enhances the effectiveness of colistin against clinical *E. coli* isolates in vitro. A proposed explanation for this synergistic effect is that eugenol interacts with the MCR‐1 protein, which is responsible for plasmid‐mediated colistin resistance [138].

Another study demonstrates that trans‐cinnamaldehyde has potent and fast‐acting antibacterial properties against Enterobacteriaceae obtained from shrimp. Furthermore, the combination of trans‐cinnamaldehyde and gentamicin showed synergistic and additive effects, indicating that plant‐based compounds such as trans‐cinnamaldehyde should be investigated as potential adjuvants to modulate and enhance the efficacy of conventional antibiotics against bacterial infections [139].

Research indicates that certain EOs can combat drug‐resistant polymicrobial biofilms. Specifically, cinnamaldehyde, carvacrol and eugenol demonstrate potent antibiofilm activity against rapidly growing mycobacteria, potentially by damaging the bacterial cell membrane. A key finding is that these EOs enhance the effectiveness of standard antibiotics (amikacin and clarithromycin); their combined use produces a synergistic effect that more effectively reduces biofilm formation and kills the bacteria [140].

The multitude of compounds within EOs target various bacterial structures simultaneously. This multitarget mechanism hinders the ability of pathogens to develop resistance. Despite this advantage, clinical application requires further investigation into several key factors [141, 142].

Several studies have demonstrated that combining EOs or EOCs with antibiotics can significantly lower the required doses of both agents while improving antimicrobial efficacy. Potential mechanisms of synergy between EOs or EOCs and antibiotics include (a) EO‐mediated membrane damage, which enhances antibiotic penetration or induces the loss of macromolecular components [143], (b) synergistic effects in reducing biofilm formation [140], (c) the multicomponent nature of EOs, which allows them to target different bacterial structures or antimicrobial pathways simultaneously, unlike many single‐target conventional antimicrobials [119, 141], (d) perforation of the bacterial surface by EOCs, thereby promoting antibiotic permeability across the lipid bilayer of the membrane [49], (e) inhibition of antibiotic‐degrading enzymes [144], (f) EOs likely work in a similar way, which explains why they are so effective together [33, 132], and (g) suppression of efflux pumps, as EOs and their volatile constituents can reverse drug resistance in Gram‐negative bacteria through this mechanism [145]. These findings establish a theoretical basis for the potential therapeutic use of these combinations.

The ability of EOs to combat microbes and block efflux pumps makes them a potential solution for reversing antibiotic resistance in bacteria [88].

Finally, the full range of modes of action for EO constituents remains unknown. Understanding these mechanisms is essential for predicting their impact on various microbes and their interactions with antibiotics and other antimicrobials. The primary challenge in using these constituents to manage bacterial infections is their frequent lack of sufficient potency when used alone. Furthermore, they can have detrimental organoleptic effects when applied at the high concentrations required for an antibacterial effect. One solution is to exploit the synergistic effects between different volatile compounds. However, the interactions that lead to antagonistic, additive or synergistic effects are still poorly understood. Clarifying these relationships could aid in developing new, more potent antimicrobial blends and in understanding how the components of crude EOs interact [68].

## 7. Delivery Strategies and Bioavailability Enhancement of EOs

### 7.1. Nanoencapsulation Strategies for Enhanced Delivery

EOs and EOCs possess significant pharmaceutical potential as both active therapeutic agents and natural permeation enhancers. However, their clinical translation is hindered by inherent physicochemical challenges, high volatility, lipophilicity, instability and poor aqueous solubility, leading to rapid metabolism, low systemic bioavailability and unreliable efficacy [146].

Advanced nanoencapsulation strategies have been developed to overcome these limitations. Key approaches include

1. Lipid‐based nanocarriers: Systems such as liposomes, nanostructured lipid carriers (NLCs) and nanoemulsions solubilize lipophilic EOs within a lipid core. This protects them from degradation and enhances absorption via lymphatic transport and mucosal permeation [147].
2. Polymeric and other nanoparticles: Polymers (synthetic and natural) and solid lipid nanoparticles (SLNs) create protective matrices that enable controlled release, mask unpleasant odours and improve stability [148, 149].
3. Microencapsulation: Nanoemulsions, microencapsulation, liposomal encapsulation, polymer nanoparticles and SLNs each provide distinct benefits. Specifically, microencapsulation techniques—such as spray drying or coacervation—can embed EOs within a protective matrix made from materials such as starch, chitosan or gelatin. This process traps the EOs, reduces their volatility and extends their shelf life, while also overcoming limitations such as instability and poor bioavailability [150].

These encapsulation methods collectively enhance dissolution, stability and bioavailability, unlocking the therapeutic potential of EOs for commercial and clinical applications.

### 7.2. Targeted Delivery Systems to Improve Bioavailability

The rational design of targeted delivery systems is crucial for enhancing the bioactivity of EOs. Nanotechnology enables the creation of particulate systems for controlled and sustained release.

1. Polymeric nanoparticles: Synthetic polymers such as poly(D, L‐lactic‐co‐glycolic acid) (PLGA) are widely used. PLGA nanoparticles are biocompatible and biodegradable and allow for high drug loading and surface modification for targeting [151]. They have effectively encapsulated agents such as thymol, improving stability and providing biphasic drug release [152, 153].
2. Chitosan‐based systems: Chitosan nanoparticles (CS NPs) offer advantages including biocompatibility, low toxicity and mucoadhesive properties due to their cationic nature [154]. Their performance depends on factors such as cross‐linking degree and particle size [155]. For example, thyme EO‐loaded CS NPs showed high encapsulation efficiency and formulation‐dependent antibacterial activity [156].
3. Cyclodextrin inclusion complexes: Cyclodextrins form host–guest complexes that shield EO molecules, addressing poor solubility and volatility. This GRAS status approach enhances stability and bioavailability and can potentiate antibacterial activity [157].

These nanodelivery systems improve dispersibility, stability and targeted delivery, offering a promising strategy to combat AMR and enhance therapeutic efficacy compared to unencapsulated EOs [157, 158].

### 7.3. Application Routes

EOs are administered via specific routes, including topical and pulmonary delivery, to leverage their properties and overcome biological barriers.

1. Topical/transdermal delivery: EOs are traditionally used for skin conditions (acne, inflammation). Advanced nanolipidic carriers such as NLCs and SLNs enhance topical delivery by increasing skin penetration, improving stability and providing sustained release, outperforming conventional formulations [159]. Permeation enhancers and elastic vesicles (e.g., ethosomes) further facilitate transport across the stratum corneum [160].
2. Pulmonary and aromatherapy delivery: Inhalation exploits the volatile nature of EOs. Pulmonary delivery of nebulized nanoformulations, including nanoemulsions, allows direct local treatment of respiratory infections, bypassing first‐pass metabolism. As an example, alkaline tea tree oil nanoemulsions represent a promising nebulizable formulation for treating bacterial pneumonia, including cases caused by drug‐resistant pathogens in an animal study [161, 162]. In aromatherapy, diffusion of citrus EOs and others is used for their perceived psychological benefits (e.g., relaxation, stress relief) [163].

Future work must advance beyond in vitro studies, focusing on in vivo pharmacokinetics, rigorous safety and toxicity profiling, clinical trials and standardization of materials and regulations to ensure effective and safe EO‐based therapies [164].

Finally, encapsulation via nano‐ and microcarriers is essential to stabilize EOs and enhance their bioavailability. Scaling up these techniques for industrial production is a key challenge. Success depends on rational material selection and process optimization, integrated with comprehensive pharmacokinetic and pharmacodynamic evaluation. This multidisciplinary approach is vital to transform volatile natural compounds into reliable, clinically effective medicines.

## 8. Conclusion

EOs fall into two main categories: terpenoids (which form the bulk) and nonterpenoids, such as phenylpropanoids. EOs are complex natural mixtures with broad antimicrobial properties, primarily attributed to oxygenated terpenoids such as phenolic terpenes, phenylpropanoids and alcohols. While some constituents (e.g., hydrocarbons) are less active by themselves, when combined, they can increase overall bioactivity. The mechanism of action of EOs or EOCs on bacteria includes (i) membrane disruption as a primary action, which increases permeability, (ii) inhibition of energy production, (iii) protein dysfunction, (iv) nucleic acid (DNA/RNA) interference, (v) efflux pump inhibition, (vi) interference with other cellular processes, such as virulence mechanisms (e.g., quorum‐sensing interference, biofilm disruption and toxin production inhibition), and (vii) synergistic effects.

The synergistic antimicrobial effect of EOs or EOCs, both in combinations with each other and with antibiotics, is well established against pathogenic and drug‐resistant microbes. Although this synergy is widely documented for many bacterial species, the specific cellular mechanisms behind it remain poorly understood. Potential mechanisms of synergy between EOs or EOCs and antibiotics include (a) EO‐mediated membrane damage, (b) synergistic effects in reducing biofilm formation, (c) the multicomponent nature of EOs or EOCs, which allows them to target different bacterial structures or antimicrobial pathways simultaneously, (d) perforation of the bacterial surface by EOCs, thereby promoting antibiotic permeability across the lipid bilayer of the membrane, (e) inhibition of antibiotic‐degrading enzymes, (f) EOs likely working in a similar way to antibiotics, which explains their effectiveness in combination, and (g) suppression of efflux pumps, as EOs and their volatile constituents can reverse drug resistance.

On the other hand, while this review has focused on the mechanisms underlying the antibacterial efficacy of EOs and EOCs, it is important to acknowledge that some affected microbial pathways have analogous components in human cells. This shared biology raises a necessary consideration for selective toxicity. The current evidence suggests that the preferential disruption of microbial systems arises from differences in cell membrane complexity, the achievement of locally high concentrations at the site of infection and the combinatorial multitarget nature of EOs, which may overwhelm bacterial infection and its virulence more readily than that of human cells. EOs possess antimicrobial and anti‐inflammatory properties that offer a promising theoretical basis for their use as complementary agents in sepsis management by targeting key pathological cascades; direct clinical evidence remains limited. Surprisingly, there is a dearth of clinical study data regarding the immunomodulatory effects of EOs. Nonetheless, the therapeutic utility and safety of EOs are fundamentally governed by concentration, exposure route and formulation. Future work aimed at clinical translation should therefore prioritize therapeutic index assessments and delivery strategies that maximize selective antibacterial action while minimizing potential off‐target effects on host cells, and significant hurdles, including delivery methods, bioavailability and standardized dosing, must be overcome before they can be reliably integrated as adjuncts to established conventional therapies such as antibiotics and supportive care.

Future research must therefore employ interdisciplinary and innovative approaches to clarify these mechanisms. A deeper understanding of how EOs and antibiotics interact at a molecular level is critical for designing effective combination therapies and unlocking their full potential. Although EOs show great promise, several challenges must be addressed in future work, including standardization (variability in EO composition based on plant source, growth conditions and extraction methods), delivery systems (development of nanoemulsions and encapsulation to improve stability and bioavailability), safety regulation, toxicity and tolerance level for use in each case.

## Funding

The authors extend their appreciation to Prince Sattam bin Abdulaziz University for funding this research work through Project Number PSAU/2025/03/34060.

## Conflicts of Interest

The authors declare no conflicts of interest.

## Data Availability

Data sharing is not applicable to this article as no datasets were generated or analysed during the current study.
